# Novel *CLCNKB* Mutation in Two Siblings With Classic Bartter Syndrome

**DOI:** 10.1155/crig/8862780

**Published:** 2025-03-04

**Authors:** Navid Roodaki, Leigh Michelle Salinas, Ebner Bon G. Maceda, Jorelyn Frias

**Affiliations:** ^1^Department of Pediatrics, Ilocos Training Regional and Medical Center, San Fernando, La Union, Philippines; ^2^College of Medicine, Mariano Marcos Memorial State University, Batac, Ilocos Norte, Philippines; ^3^Center for Human Genetics Services, Institute of Human Genetics, National Institutes of Health, University of the Philippines Manila, Manila, Philippines

**Keywords:** bartter syndrome, CLCNKB, novel mutation

## Abstract

**Background:** Bartter syndrome is a rare genetic illness characterized by impairment in kidney function caused by different gene defects. The primary pathogenic mechanism of Bartter syndrome is defective salt reabsorption, predominantly in the thick ascending limb of the loop of Henle.

**Case Presentation:** Here, we present a case series between 2 siblings diagnosed with Bartter syndrome through clinical and genetic analyses. Both patients presented with severe dehydration secondary to polyuria which caused persistent electrolyte imbalances. However, the second sibling presented with hydrocephalus which may be associated with Bartter Syndrome. Genetic analysis determined the presence of a known pathogenic mutation and a novel mutation in the CLCNKB variant.

**Conclusions:** Bartter syndrome Type III is a genetic disorder that must be identified clinically without delay, as it typically manifests as acute dehydration due to polyuria and vomiting. Hydrocephalus, although cannot be concluded to be a complication of Bartter syndrome, can be associated due to several electrolyte imbalances involved in this condition. Genetic testing is essential for identifying unidentified pathogenic variants that will aid future patients diagnosed with this condition. Genetic counseling is of the utmost importance for these families affected by the condition in question.

## 1. Background

Bartter syndrome (BS) exemplifies the challenges of managing inherited disorders, especially those affecting the kidneys. This syndrome causes low potassium levels, metabolic alkalosis (imbalance in the body's acid–base level), high levels of renin and aldosterone hormones, and loss of salt, all while blood pressure remains normal. It affects about 1.2 in every million people, making it tough to diagnose and treat quickly, especially due to limited testing resources. Novel gene mutations, like the one identified in this case series, play a crucial role in understanding the underlying genetic mechanisms of rare diseases such as BS. Identifying these mutations not only aids in accurate diagnosis but also opens avenues for targeted therapies and potential advancements in medical management. Here, we discuss the case of two siblings diagnosed with BS Type III, highlighting a new gene mutation as the cause of their condition.

## 2. Case Presentation

### 2.1. Case 1

The first child is a 4-year-old boy (Sibling A), who came in due persistent vomiting and weakness.

At 2 months of age, he was admitted to a tertiary hospital due to continuous vomiting. The patient apparently well until the day prior to admission; he experienced more than seven episodes of recurrent bouts of nonbilious, nonprojectile vomiting of previously consumed milk with concomitant retching, accompanied by five episodes of mucoid loose watery stool, nonbloody, nonfoul smelling. The patient was brought to the emergency department due to the persistence of symptoms. Prenatal, natal, and neonatal history were noncontributory.

Upon assessment, the patient was severely wasted and severely stunted, weak-looking and only responded to pain with poor skin turgor. The patient was tachycardic at 165 cpm, tachypneic at 59 bpm, and afebrile. Random blood sugar was low at 14 mg/dL. Immediate fluid resuscitation was performed resulting in an improvement in the patient's vital signs and hydration status.

The complete blood count was normal, while the urinalysis revealed hyposthenuria. Serum electrolytes showed hypokalemia at 2.3 mmol/L, hyponatremia at 123 mmol/L, hypochloremia at 85 mmol/L, normal ionized calcium at 1.17 mmol/L, and normal magnesium at 1.00. Fluid replacement and electrolyte correction were performed; however, it was persistent on the subsequent hospital days. Polyuria was also noted at 15.7 cc/kg/hour during admission. Considerations were nephrogenic diabetes insipidus, renal tubular acidosis, Gittleman, and BS. Another laboratory test to narrow down the diagnosis included arterial blood gas, which revealed metabolic alkalosis, and kidney ureter and bladder (KUB) ultrasound showed bilateral nephrocalcinosis. In addition, the aldosterone/plasma renin activity ratio was high at 68 ng/dL; thus BS was highly considered. Hence, blood samples for gene analysis were sent to Invitae.

### 2.2. Case 2

The second child, a 3-year-old girl (Sibling B), who was severely wastered and severely stunted, was brought the emergency department at 7 of age due to severe dehydration. She had exactly the same presentation as his the emergency department at 7.

Laboratory tests showed hypokalemia at 2.2 mmol/L, hyponatremia at 126.6 mmol/, hypochloremia at 70.6 mmol/L, normal ionized calcium at 1.23 mmol/L, and normal magnesium at 1.05. Creatinine and blood urea nitrogen were normal, urinalysis revealed hyposthenuria with a specific gravity of 1.005, and complete blood count was normal. The aldosterone/plasma renin activity ratio was increased at 75 ng/dL. Arterial blood gas showed metabolic alkalosis. Hence, inherited renal tubulopathies were again considered. On subsequent hospitalizations, the patient had generalized tonic-clonic seizures lasting at least 2 minutes; bacterial meningitis was suspected at this time. An emergency contrast-enhanced cranial CT scan was performed which showed noncommunicating hydrocephalus and no meningeal enhancement; hence, ventriculoperitoneal shunt placement was performed.

### 2.3. Gene Variant Analysis

Both specimens were submitted together with samples from both parents for carrier screening. As seen in Table 1, each of the parent was a carrier of the *CLCNKB* gene variant. The father carried a known pathogenic variant caused by a missense mutation in which the base cytosine (C) is changed to thymine (T) (c.1312C < T). This substitution leads to a change in the amino acid sequence (p.Arg438Cys) where arginine, which is basic and polar, is changed to cysteine, which is neutral and slightly polar, at codon 438 of the *CLCNKB* protein. This variant disrupts the p.Arg438 amino acid residue in CLCNKB. This suggests that this residue is clinically significant, and that variants that disrupt this residue are likely to be disease-causing. The mother on the other hand carried a *CLCNKB* gene variant with a duplication of six bases (c.987_992dup) causing an insertion of an additional leucine and an alanine (p.Leu330_Ala331dup). This variant, c.987_992dup, results in the insertion of 2 amino acid(s) of the CLCNKB protein (p.Leu330_Ala331dup) but otherwise preserves the integrity of the reading frame. For this reason, this variant has been classified as an uncertain significance. Despite this classification, one causative variant is insufficient as an explanation for the individual's condition because BS is an autosomal recessive disorder. The DNA analysis showed that Sibling A and Sibling B had both inherited the known pathogenic *CLCNKB* gene variant from the father as well as the novel *CLCNKB* gene variant from the mother.

### 2.4. Outcome and Follow-Up

Both patients were eventually discharged and improved despite persistent electrolyte imbalances. They were both advised monthly follow-up with monthly laboratory test monitory to ensure that all laboratory test. Both patients were also referred for nutritional upbuilding.

## 3. Discussion and Conclusions

### 3.1. Discussion

We presented 2 cases of BS among siblings with similar manifestations of severe dehydration due to excessive polyuria and laboratory findings.

BS is a known autosomal recessive disorder of the renal tubules caused by mutations in genes encoding ion channels and/or transporters. This condition was first discovered in 1962 by Bartter et al. [1, 2] and was then subclassified into 5 different forms based on the type of gene mutation, with BS III (*CLCNKB*) being the most prevalent [3]. Most cases of BS III exhibited in early to late childhood; however, some cases present at an earlier age with more severe symptoms [4].

Type III BS is known to have the mildest symptoms of all subtypes. According to the medical literature, the most common clinical manifestations are polyuria, polydipsia, clinical indicators of hypovolemia due to dehydration, failure to thrive, and poor growth [4, 5]. All of which are present in our cases.

Laboratory tests (Table 2) in patients invariably reveal electrolyte imbalances, including hypokalemia, hypochloremia, and hyponatremia [6, 7], which are present in both instances. These findings in BS stem down from a defect in the ion transport system [5, 7]. The transport of sodium (Na^+^), potassium (K^+^), and chloride (Cl^−^) ions across cell membranes into the cytoplasm alters the intracellular ion balance, and thus, in order to maintain homeostasis, they must be relocated either to the interstitium or to the tubular lumen [8]. Therefore, Na^+^ is actively pumped out of TAL by Na^+^K^+^ATPase and K^+^ is recycled to the tubular lumen by the renal outer medullary K^+^ (ROMK) channel [4, 8, 9]. ROMK is encoded by the *KCNJ1* gene and is localized at the luminal surface of the epithelial cells. Cl^−^ can exit the cell via the two known basolateral chloride channels *ClC-Ka* and *ClC-Kb* (encoded by the *CLCNKA* and *CLCNKB* genes) [9]. The presence of the Barttin subunit, encoded by the *BSND* gene, is required by both these chloride channels for normal function as the subunit plays a key role in channel integrity, efficient membrane transport, and channel gating [8–11]. The *ClC-Ka* is predominantly expressed in the thin limb of the loop of Henle, and the *ClC-Kb* in the TAL, distal convolute tubule (DCT), and in collecting duct-intercalated cells. *ClC-Ka* and *ClC-Kb*, together with the Barttin subunit, are also expressed in the inner ear and contribute to the production of endolymph [8–13]. In both cases, further laboratory findings include increased renin and aldosterone levels and reduced urine osmolality [7–9].

Imaging is of minimal value for BS; however, if patients undergo renal ultrasonography, they typically find nephrocalcinosis, as was the case with our patients [7, 8, 12]. It is unknown what causes nephrocalcinosis, however, it is believed that hypercalciuria and hyperuricosuria have a role [8]. The presence of hydrocephalus is an unusual finding in Sibling B. In textbooks, there is no association between BS and hydrocephalus, although Ozdemir et al. reported a case of neonatal BS presenting with cholelithiasis and hydrocephalus. Although there is no known pathophysiology that links hydrocephalus to BS, it is speculated that ion transport in the central nervous system may play a role in causing this complication; however, additional research and similar cases should be reported prior to drawing any further conclusions [10].

Growth failure was also observed in both siblings, as is typical for BS III. A case study by Colussi et al. focuses on the association between growth hormone deficiency and postulated possible associated [7]. Similarly, Flyvbjerg et al. postulated the same association but directed hypokalemia as the cause of growth hormone deficiency among tubular disorders [8]. However, there is currently no consensus regarding the determination of GH levels among these patients. It is still a case to case basis.

As BS Type III is a known autosomal recessive disorder, investigation among family parents is warranted. The classic BS variant is caused by mutations in the *CLCNKB* gene located on 1p36, which encodes the basolaterally located renal chloride channel *ClC-Kb,* which mediates Cl-efflux from epithelial cells lining the TAL and DCT [11, 14–16]. Thus, inactivating mutations in *CLCNKB* affect the basolateral exit of chloride which in turn reduces the reabsorption of NaCl in the TAL and DCT. This explained the frequent hyponatremia, hypokalemia, and metabolic alkalosis that are present in our patients [3, 17].

In this instance, both siblings inherited a missense mutation plus a frameshift mutation. In addition, a new mutation designated VUS was identified in the mother's *CLCNKB* gene. However, this mutation is not yet recorded in the BS III database, but based on the clinical presentation of both siblings, it is most likely pathogenic and will be reclassified. A similar finding from a case report by Tian et al. discussed a novel heterozygous mutation in a late onset BS Type II [13]. In a separate case report, a new mutation in the *CLCNKB* gene was associated with growth retardation in BS Type III patients [13, 18–20]. In light of this, genetic counseling should be provided to all patients with BS as well as parents of affected children. As an autosomal recessive condition, at-risk couples (both persons are carriers of a disease-causing mutation) have a 25% chance per pregnancy of having an affected kid [13].

The treatment of BS is directed toward the specific symptoms that are presented by the individual patient. The mainstay of treatment is restoring the proper balance of fluids and electrolytes in the body [13, 21]. Supplementation with sodium chloride constitutes a physiologic treatment that can support extracellular volume and improve electrolyte abnormalities and at least 5–10 mmol/kg/d sodium chloride has been recommended [2, 11]. If potassium is supplemented, potassium chloride should be used. Potassium salts (e.g., citrate) should be avoided because they could potentially worsen the metabolic disturbance by aggravating alkalosis. 

Pharmacologic suppression of prostaglandin formation addresses the underlying pathophysiology, and multiple clinical observational studies have shown it to be beneficial in the form of improved growth and electrolyte profiles. The use of selective COX-2 inhibitors has also been reported in BS because it can reduce the glomerular filtration rate, thereby further reducing renal salt and water losses. Commonly used NSAIDs in BS are indomethacin (1–4 mg/kg/d divided in 3-4 doses), ibuprofen (15–30 mg/kg daily in 3 doses), and celecoxib (2–10 mg/kg/d in 2 doses) [11, 13, 22].

Treatment and follow-ups should be tailored to the patient on the basis of clinical manifestation, medical history, stage of development, molecular defect, and the clinician's expert judgment in close contact with the patient's local health care provider. The prognosis is good if treatment is started at an early stage [20].

### 3.2. Conclusion

Patients with BS III manifest later in life and may be asymptomatic or mildly symptomatic. Initial symptoms include vomiting and polyuria, which result in dehydration and electrolyte imbalance. Mutations in the *CLCNKB* gene which codes for the chloride channel *CLC-Kb* cause classic BS. The *CLC-Kb* protein mediates Cl-efflux from the distal epithelial cell to the interstitium. During the first year of life, the majority of patients experience hypokalemia, hypochloremia, and failure to thrive. Replacement of fluids and electrolytes is the cornerstone of treatment. With early treatment, the prognosis is favorable. As BS is a known inherited disorder, various gene mutations, known and unknown can be identified in each patient which warrants genetic testing and counseling [6].

## Figures and Tables

**Table 1:** Gene variant analysis result of both parents and siblings.

Carrier	Gene	Variant	Zygosity	Variant
Mother	CLCNKB	c.987_992dup (p.Leu330_Ala331dup)	Heterozygous	Uncertain significance

Father	CLCNKB	c.1312C > T (p.Arg438Cys)	Heterozygous	Pathogenic

Sibling 1	CLCNKB	c.987_992dup (p.Leu330_Ala331dup)	Heterozygous	Uncertain significance
	CLCNKB	c.1312C > T (p.Arg438Cys)	Heterozygous	Pathogenic

Sibling 2	CLCNKB	c.987_992dup (p.Leu330_Ala331dup)	Heterozygous	Uncertain significance
	CLCNKB	c.1312C > T (p.Arg438Cys)	Heterozygous	Pathogenic

**Table 2:** Clinical data of both siblings upon admission.

Clinical data	Case 1	Case 2
Gender	Male	Female
Age (years)	4-year-old	3-year-old
Weight (kg)	8 kg	6.3 kg
Urine output on admission	15.7 cc/kg/hr	14 cc/kg/hr
Urine specific gravity on admission	1.100	1.005
Electrolytes (mmol/L)		
• Potassium (3.5–5)	2.3	2.2
• Sodium (135–148)	123	126.6
• Chloride (96–107)	85	70.6
• Ionized calcium (1.120–1.320)	1.17	1.23
• Magnesium (0.85–1.10)	1.00	1.05
Aldosterone/plasma renin ratio (ng/dL)	68	75
Arterial blood gas		
• pH	7.55	7.70
• pCO_2_	40	48
• HCO_3_	35	36
• Interpretation	Uncompensated metabolic alkalosis	Partially compensated metabolic alkalosis
KUB ultrasound	Bilateral nephrocalcinosis	Bilateral nephrocalcinosis

## Data Availability

The data that support the findings of this study are available in this manuscript. Original laboratory results may be given upon request of the reviewer.

## References

[1] Konrad M., Nijenhuis T., Ariceta G. (2021). Diagnosis and Management of Bartter Syndrome: Executive Summary of the Consensus and Recommendations From the European Rare Kidney Disease Reference Network Working Group for Tubular Disorders. *Kidney International*.

[2] Konrad M., Vollmer M., Lemmink H. H. (2000). Mutations in the Chloride Channel Gene CLCNKB as a Cause of Classic Bartter Syndrome. *Journal of the American Society of Nephrology*.

[3] Kurtz I. (1998). Molecular Pathogenesis of Bartter’s and Gitelman’s Syndromes. *Kidney International*.

[4] Kliegman R., Stanton B., Behrman R. E., Geme J. W., Schor N. F., Nelson W. E. (2019). *Nelson Textbook of Pediatrics*.

[5] Nascimento C. L., Garcia C. L., Schvartsman B. G., Vaisbich M. H. (2014). Treatment of Bartter Syndrome. Unsolved Issue. *Jornal de Pediatria*.

[6] Bartter Syndrome. NORD (National Organization for Rare Disorders). https://rarediseases.org/rare-diseases/bartters-syndrome/.

[7] Colussi G., De Ferrari M. E., Tedeschi S., Prandoni S., Syrén M. L., Civati G. (2002). Bartter Syndrome Type 3: An Unusual Cause of Nephrolithiasis. *Nephrology Dialysis Transplantation*.

[8] Flyvbjerg A., Dørup I., Everts M. E., Orskov H. Evidence That Potassium Deficiency Induces Growth Retardation Through Reduced Circulating Levels of Growth Hormone and Insulin-Like Growth Factor I. *Metabolism*.

[9] Özdemir Ö. M. A., Çıralı C., Yılmaz Ağladıoğlu S., Evrengül H., Tepeli E., Ergin H. (2016). Neonatal Bartter syndrome With cholelithiasis and hydrocephalus: Rare association. *Pediatrics International*.

[10] Colussi G., De Ferrari M. E., Tedeschi S., Prandoni S., Syrén M. L., Civati G. (2002). Bartter Syndrome Type 3: An Unusual Cause of Nephrolithiasis. *Nephrology Dialysis Transplantation*.

[11] Avner E. D., Harmon W. E., Niaudet P., Yoshikawa N., Emma F., Goldstein S. (2020). *Pediatric Nephrology*.

[12] Yang X., Zhang G., Wang M., Yang H., Li Q. (2018). Bartter Syndrome Type 3: Phenotype-Genotype Correlation and Favorable Response to Ibuprofen. *Frontiers in Pediatrics*.

[13] Tian M., Peng H., Bi X. (2022). Late-Onset Bartter Syndrome Type II Due to a Novel Compound Heterozygous Mutation in *KCNJ1* Gene: A Case Report and Literature Review. *Frontiers of Medicine*.

[14] Padilla C. D., de la Paz E. M. C. Genetic Services and Testing in the Philippines. *Journal of community genetics*.

[15] Yang X., Zhang G., Wang M., Yang H., Li Q. (2018). Bartter Syndrome Type 3: Phenotype-Genotype Correlation and Favorable Response to Ibuprofen. *Frontiers in Pediatrics*.

[16] Mali L. P., Meena S. S., Baniwal M. (2015). Classical Bartter Syndrome—A Case Report. *Indian Journal of Medical Specialities*.

[17] Francini F., Gobbi L., Ravarotto V. (2021). The Dietary Approach to the Treatment of the Rare Genetic Tubulopathies Gitelman’s and Bartter’s Syndromes. *Nutrients*.

[18] Kömhoff M. (2011). Acetyl Salicylic Acid Treatment in Neonatal Bartter Syndrome—A Commentary Letter. *Pediatric Nephrology*.

[19] Wu X., Yang G., Chen S. (2020). Bartter Syndrome With Long-Term Follow-Up: A Case Report. *Journal of International Medical Research*.

[20] INSERM Orphanet: Bartter Syndrome Type 3. https://www.orpha.net/consor/cgi-bin/OC_Exp.php?lng=en&Expert=93605.

[21] Tda C., Heilberg I. P. (2018). Bartter Syndrome: Causes, Diagnosis, and Treatment. *International Journal of Nephrology and Renovascular Disease*.

[22] Brambilla I., Poddighe D., Mantelli S. S., Guarracino C., Marseglia G. L. (2018). Bartter Syndrome and GH Deficiency: Three Siblings With a Novel CLCNKB Mutation. *Pediatrics International*.

